# A Core Genome Multilocus Sequence Typing Scheme for *Pseudomonas aeruginosa*

**DOI:** 10.3389/fmicb.2020.01049

**Published:** 2020-05-26

**Authors:** Romário Oliveira de Sales, Letícia Busato Migliorini, Renato Puga, Bela Kocsis, Patricia Severino

**Affiliations:** ^1^Hospital Israelita Albert Einstein, Albert Einstein Research and Education Institute, São Paulo, Brazil; ^2^Institute of Medical Microbiology, Semmelweis University, Budapest, Hungary

**Keywords:** *Pseudomonas aeruginosa*, core genome multilocus sequence typing, sequence type, genome sequencing analysis, mlst

## Abstract

*Pseudomonas aeruginosa* is a ubiquitous microorganism and an important opportunistic pathogen responsible for a broad spectrum of infections mainly in immunosuppressed and critically ill patients. Molecular investigations traditionally rely on pulsed field gel electrophoresis (PFGE) and multilocus sequence typing (MLST). In this work we propose a core genome multilocus sequence typing (cgMLST) scheme for *P. aeruginosa*, a methodology that combines traditional MLST principles with whole genome sequencing data. All publicly available complete *P. aeruginosa* genomes, representing the diversity of this species, were used to establish a cgMLST scheme targeting 2,653 genes. The scheme was then tested using genomes available at contig, chromosome and scaffold levels. The proposed cgMLST scheme for *P. aeruginosa* typed over 99% (2,314/2,325) of the genomes available for this study considering at least 95% of the cgMLST target genes present. The absence of a certain number gene targets at the threshold considered for both the creation and validation steps due to low genome sequence quality is possibly the main reason for this result. The cgMLST scheme was compared with previously published whole genome single nucleotide polymorphism analysis for the characterization of the population structure of the epidemic clone ST235 and results were highly similar. In order to evaluate the typing resolution of the proposed scheme, collections of isolates belonging to two important STs associated with cystic fibrosis, ST146 and ST274, were typed using this scheme, and ST235 isolates associated with an outbreak were evaluated. Besides confirming the relatedness of all the isolates, earlier determined by MLST, the higher resolution of cgMLST denotes that it may be suitable for surveillance programs, overcoming possible shortcomings of classical MLST. The proposed scheme is publicly available at: https://github.com/BioinformaticsHIAEMolecularMicrobiology/cgMLST-Pseudomonas-aeruginosa.

## Introduction

*Pseudomonas aeruginosa* is an opportunistic human pathogen responsible for nosocomial infections worldwide, such as ventilator-associated pneumonia and burn wound infections, mainly in immunosuppressed patients (De Bentzmann and Plesiat, 2011; Gellatly and Hancock, 2013; Cabot et al., 2016; Sousa et al., 2018; Yin et al., 2018). Moreover, it is highly involved in chronic pneumonia in patients with cystic fibrosis (CF) (Lyczak et al., 2000; Breidenstein et al., 2011; Ballarini et al., 2012; Lopez-Causape et al., 2017). Intrinsic and acquired antibiotic resistance limits treatment of these infections (Gellatly and Hancock, 2013; Gomila et al., 2013; Arzanlou et al., 2017). Also, due to its ability to form biofilm, *P. aeruginosa* can remain viable for a long time on hospital surfaces and medical fomites, contributing to its spread in clinical settings and possible outbreaks (Lister et al., 2009; Veesenmeyer et al., 2009; Breathnach et al., 2012; Gill et al., 2016; Alipour et al., 2017; Moradali et al., 2017). As such, *P. aeruginosa*, especially those belonging to high-risk clonal complexes which are often resistant, are a threat to public health worldwide (Woodford et al., 2006; Mulet et al., 2013; Treepong et al., 2018).

High-resolution sequence typing is important to evaluate epidemiological links and to characterize possible transmission sources during outbreaks. Currently, two major subtyping techniques are used to identify the clonal relationship between *P. aeruginosa* isolates: Pulsed-field Gel Electrophoresis (PFGE) and multilocus sequence typing (MLST) (Curran et al., 2004; Johnson et al., 2007; Kidd et al., 2011). PFGE is one of the most useful DNA fingerprinting techniques and a method widely used in hospitals to identify *P. aeruginosa* outbreaks. However, PFGE is a time-consuming and laborious method, and often not easily reproducible between clinical laboratories (Tenover et al., 1995; Fothergill et al., 2010; Lila et al., 2018; Martak et al., 2019). In contrast, MLST, developed in 2004 for *P. aeruginosa*, is based on the sequencing and evaluation of the allelic variation of seven housekeeping genes, generating the so-called sequence types (STs) to characterize isolates (Curran et al., 2004). However, the discriminatory power of MLST does not always allow enough resolution during outbreaks. Therefore, both PFGE and MLST methods are often simultaneously required (Li et al., 2009; Kidd et al., 2011; Sabat et al., 2013).

Recently, methods based on whole genome sequencing (WGS) have become viable due to lower sequencing costs; enabling high discrimination between isolates in epidemiological investigation. Nevertheless, suitable bioinformatics tools are necessary to handle and interpret sequence data (Tang et al., 2017; Bogaerts et al., 2019). The aim of Core Genome MLST (cgMLST) is to extend the MLST concept to a larger number of genes of the core genome. It relies on WGS but allows for the examination of a fixed number of genome *loci* and the creation of a systematic allele numbering system. cgMLST schemes may be developed and locally implemented using commercial softwares such as BioNumerics (Applied Maths, Sint-Martens-Latem, Belgium), SeqSphere+ (Ridom, Münster, Germany), and BIGSdb^1^ (Schürch et al., 2018). The design may lead to maximum resolution for both epidemiological and surveillance analyses (Mellmann et al., 2011; de Been et al., 2015; Zhou et al., 2017; Neumann et al., 2019). The cgMLST has already shown good results for bacterial pathogens such as *Mycobacterium tuberculosis*, *Acinetobacter baumannii*, *Klebsiella pneumoniae*, and *Enterococcus faecalis* (Kohl et al., 2014; Higgins et al., 2017; Zhou et al., 2017; Neumann et al., 2019).

Publicly available cgMLST schemes for *P. aeruginosa* do not exist yet. Two *ad hoc* schemes have been constructed using SeqSphere+ (Ridom, Münster, Germany) but they have not been validated or used outside the context proposed by the authors (Mellmann et al., 2016; Royer et al., 2020). In this study, we created a cgMLST scheme for *P. aeruginosa* using the open source ChewBBACA algorithm (Silva et al., 2018). The scheme was created taking in consideration the core genome of all available *P. aeruginosa* genome sequences at the time of this publication and it is freely available^2^. We evaluated this scheme for its ability to discriminate isolates within the same STs (ST146, ST235, and ST274), both in an outbreak setting and using unrelated isolates. Taken together our results indicate that the scheme may be adequate for epidemiology and surveillance approaches and outbreak investigations and we aim to foment discussions and possibly help in establishing a cgMLST consensus for *P. aeruginosa*.

## Materials and Methods

### Core Genome Multilocus Sequence Typing Scheme

A *P. aeruginosa* cgMLST scheme was set up with the Comprehensive and Highly Efficient Workflow for a Blast Score Ratio Based Allele Calling Algorithm (ChewBBACA) (Silva et al., 2018). Completed genome sequences (FASTA files) of *P. aeruginosa* publicly available at the NCBI Reference Sequence database (RefSeq)^3^ in September 2018 were used (Supplementary Table S1). A total of 141 complete sequences were annotated with *Prodigal v2.6.3* (Hyatt et al., 2010) in the first step of the algorithm (*CreateSchema*). The genome sequence of *P. aeruginosa* PAO1 (RefSeq assembly accession: GCF_000006765.1) was used only as the reference genome to predict the wgMLST *loci* and then removed from cgMLST analysis. Briefly, in the first step, the algorithm defined coding sequences (CDs) for each genome, compared them in a pairwise way and generated a single FASTA file containing the selected CDs. In the next step, the allele-calling algorithm (*AlleleCall*), identified and excluded possible paralogous *loci*. The remaining list of *loci*, now called the wgMLST, was then used to define the cgMLST scheme. We chose to select candidate *loci* for the cgMLST scheme present in 100% of the available complete genomes (141 genomes). This choice minimizes the exclusion of core genome *loci* due to misassembling in incomplete unfinished sequences. In the validation step, we lowered the stringency and kept candidate *loci* common to 99% of the isolates. Since the sequences used during this step were unfinished genomes, and due to the genetic plasticity of *P. aeruginosa*, we believe this new cut-off was necessary and sufficient to make the typing scheme suitable for most isolates of various origins. Publicly available unfinished genomes of *P. aeruginosa* (contigs, scaffold, or chromosome level genome sequences) were considered for the validation of the cgMLST scheme (see text footnote 3) (Supplementary Table S1). In order to ensure sufficient quality of the sequence data, all genomes for which no MLST could be assigned using the sanger-pathogens/mlst_check script^4^ ^,5^ (J. Page et al., 2016) were filtered out. Additionally, unfinished genomes with ≥200 contigs were not included. New STs were assigned when no correspondent STs were found in the pubMLST database for the purpose of this study. All the steps followed for the creation of the cgMSLT scheme as well as input and output files are available at GitHub https://github.com/BioinformaticsHIAEMolecularMicrobiology/cgMLST-Pseudomonas-aeruginosa.

### Graphic Representation of cgMLST Results: Minimum-Spanning Trees

Using the allelic profiles obtained by the cgMLST scheme for each isolate, minimum-spanning trees were constructed using the software GrapeTree (version 1.5.0) with parameters implemented in MSTree v2 ignoring missing values for the entire collection. Furthermore, similarity trees were calculated using the neighbor-joining algorithm (StandardNJ) with default parameters implemented in GrapeTree (version 1.5.0) (Zhou et al., 2018). Trees were visualized using Interactive Tree Of Life (iTOL, version 4.2.3) (Letunic and Bork, 2016).

### cgMLST Analysis Compared With Core Genome SNP-Based Analysis for ST235

All publicly available genomes of *P. aeruginosa* ST235 were selected (128 isolates, Supplementary Table S1). These were collected over a 28-year period and came from different locations: Africa (*n* = 2), Asia (*n* = 11), Europe (*n* = 36), North America (*n* = 48), South America (*n* = 16), Oceania (*n* = 3), and unknown origin (*n* = 12). An unrooted similarity tree based on cgMLST targets was built using the neighbor-joining algorithm (StandardNJ) with default parameters implemented in GrapeTree (version 1.5.0) (Zhou et al., 2018). The resulting similarity tree from the cgMLST-based approach was visualized by iToL (version 4.2.3) (Letunic and Bork, 2016). For the core genome SNP-based analysis, the core genome was aligned using Parsnp, part of the Harvest software package (Treangen et al., 2014), using NCGM2.S1 (RefSeq assembly accession: GCF_000284555.1) as the reference genome. All sequences near the end of contigs (near possible gaps) were excluded for this analysis in order to avoid using false SNPs for similarity reconstruction (default settings in Parsnp). This tree was also visualized with iTOL. The concordance of the two methods, cgMLST and core genome SNP-based analysis, was discussed based on similarities and differences in clustering (similarity tree).

### Typing Resolution of the Proposed cgMLST-Based Analysis

Two STs were selected for comparing the typing resolution between MLST and our cgMLST scheme: ST146 and ST274. Forty-eight isolates belonging to ST146, 40 of which isolated from a single patient in United Kingdom in 2009 (Williams et al., 2015) (NCBI Bioproject PRJEB6642) and 8 unrelated isolates reported in the United Kingdom (*n* = 5) and Canada (*n* = 3) were selected. For isolates belonging to ST274, 197 isolates, most of which (*n* = 167) collected from 12 sputum specimens obtained over a 1-year period from a single patient in Canada (Diaz Caballero et al., 2015) (NCBI Bioproject PRJNA282164), and 30 unrelated ST274 isolates coming from the United States (*n* = 14), Denmark (*n* = 4), France (*n* = 2), Spain (*n* = 1), Germany (*n* = 2), Brazil (*n* = 1), China (*n* = 1), Canada (*n* = 1), and from unknown origin (*n* = 4), were included.

We also used 4 isolates of ST235 *P. aeruginosa* (NCBI Bioproject PRJEB32170) involved in a local outbreak of extended-spectrum β-lactamase SHV2a-producing *P. aeruginosa* ST235 (Royer et al., 2020) to investigate the performance of the cgMLST scheme proposed here during an outbreak setting. In the work, the authors developed an *ad hoc* cgMLST scheme using SeqSphere + software (Ridom, Münster, Germany) to evaluate the ST235 isolates involved in the outbreak and we compared the result of our scheme to theirs.

A description of isolates from bioprojects NCBI PRJEB6642, PRJNA282164, and PRJEB32170 used in this section can be found in Supplementary Table S2.

## Results

### Characterization of the *P. aeruginosa* Population Used in This Study

**FIGURE 1:**
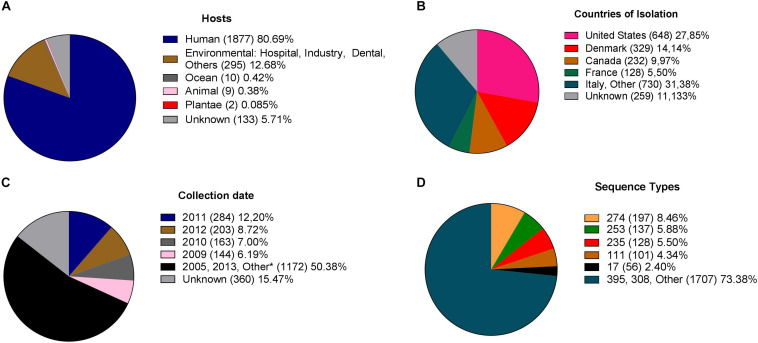
Diversity of *P. aeruginosa* isolates included in this study. **(A)** Hosts or sites of sampling. **(B)** Global distribution of the available genomes. **(C)** Temporal distribution of isolates.*Others: comprise isolates reported between 1938 and 2018 and unknown. **(D)** Distribution of sequence types as determined by MLST.

In total, 2,901 genome sequences were available at the RefSeq database. Of these genomes, 73 were not included due to the absence of MLST *loci* and 502 because they contained ≥200 contigs. Thus, from the 2,901 genomes originally available, 2,326 genomes were included in this study. Most isolates were collected from human sources (Figure 1A). They were distributed across 54 countries (Figure 1B), and the period of sampling extended across 80 years (Figure 1C).

A total of 518 different STs characterized the available population: 402 STs previously reported in the pubMLST database and 116 new STs determined for the purpose of this study (Supplementary Table S1). The most frequent STs were ST274 (*n* = 197), due to a specific project (NCBI Bioproject PRJNA282164, Supplementary Table S2) that contributed with 167 isolates, ST253 (*n* = 137) and ST235 (*n* = 128) (Figure 1D).

Among the 2,326 MLST-typed genome sequences, 141 were considered complete genomes and were considered for the initial selection of candidate targets for the cgMLST scheme. They comprised 72 distinct STs (Supplementary Table S1).

**FIGURE 2:**
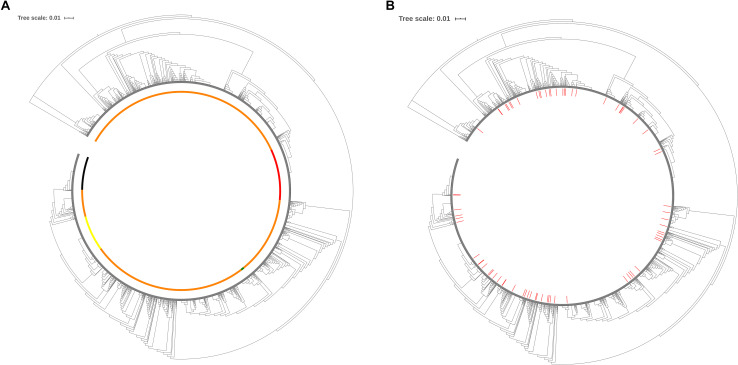
Similarity tree of 518 sequence types available for this study. **(A)** Similarity tree highlighting the most frequent STs found in the *P. aeruginosa* population available of this study. Yellow, ST253; red, ST274; black, ST235; green, ST549: clade of the PAO1; orange, all other STs. **(B)** Distribution of STs used for the cgMLST scheme development. The 72 STs that were used to define the candidate genes of the cgMLST scheme are identified in red. In green we depict 516 new STs typed and included in this study.

To evaluate the distribution of the isolates selected to create the cgMLST scheme within the *P. aeruginosa* population with publicly available sequenced genomes, maximum-likelihood similarity trees based on the concatenated conserved sequences used for MLST were calculated using FastTree 2 (v. 2.1.7, Jukes-Cantor_CAT model) (Price et al., 2010). The analysis of the tree for the total population of 2,326 genomes (518 distinct STs) (Figure 2A) and the distribution of the 141 genomes (72 STs) used for the creation of the cgMLST scheme (Figure 2B) indicate that the selected isolates reflect the diversity of the *P. aeruginosa* population with genome sequences currently available in RefSeq.

### *P. aeruginosa* cgMLST Scheme

**FIGURE 3:**
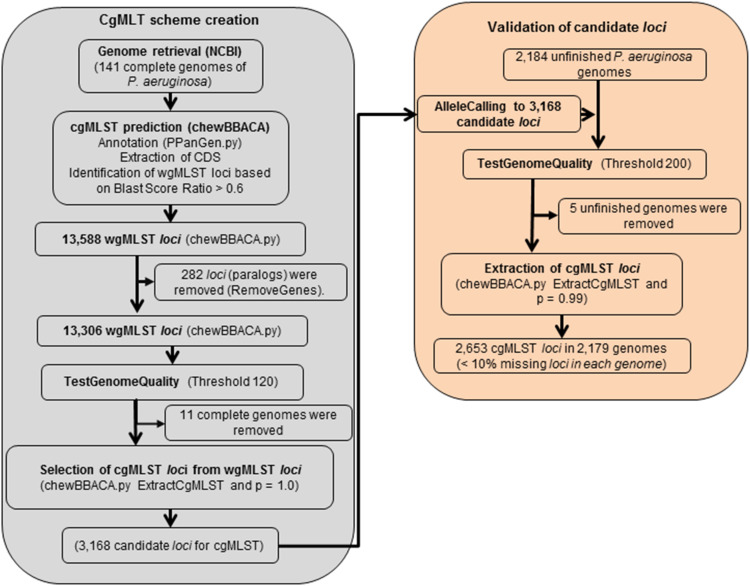
Detailed flowchart describing the development of the cgMLST scheme for *P. aeruginosa* using ChewBBACA (https://github.com/B-UMMI/chewBBACA).

For the scheme creation (Figure 3), the 141 complete genome sequences were retrieved and, using PAO1 as the reference genome (with 5,570 predicted ORFs) (Stover et al., 2000), a total of 13,588 target *loci* were annotated and generated the wgMLST data set. Following filtering steps, 282 *loci* were considered paralogous and discarded and genome quality test filtered-out additional 10,138 *loci* targets. A total of 3,168 gene targets were retained as candidates for the cgMLST scheme and they were present in 130 complete genome sequences (Supplementary Figure S1). For the evaluation step, 2,184 additional unfinished genome sequences were retrieved from the RefSeq database (Supplementary Table S1), thoroughly filtered following the same steps used for the scheme creation and analyzed using the candidate list of 3,168 gene targets. Following this step, a cgMLST scheme consisting of 2,653 gene targets (Supplementary Figure S2 and Supplementary Table S3) was defined, covering 47.63% of the 5,570 ORFs predicted for the reference strain PAO1. At least 90% of this cgMLST target list was found in 99% of all queried unfinished *P. aeruginosa* genomes. During the creation of the scheme, 11 complete genomes (fully assembled) had been discarded because they did not harbor the 3,168 candidate *loci*, and another 5 unfinished genomes were removed in the validation step. We reanalyzed those 16 isolates considering the final set of 2,653 loci and obtained similar results: with the exception of one isolate, all of the genomes contained at least 90% of the *loci*.

Finally, it has been proposed that, ideally, 95% of the cgMLST gene targets should be present is the great majority of isolates tested so that it is considered well-defined (de Been et al., 2015; Ruppitsch et al., 2015; Ghanem and El-Gazzar, 2018). The isolate could then be considered as “typed” by the cgMLST scheme. We verified our results and, considering the 2,325 genomes (141 fully assembled sequences and 2,184 unfinished genomes) only 11 isolates did not harbor at least 95% of the selected *loci* (1 fully assembled genome and 10 unfinished genome sequences). Genome sequence quality is possibly the main reason for the exclusion of the unfinished genome sequences, but for the fully assembled sequence, specific characteristics of the isolate would have to be analyzed in order to understand this result.

In summary, the proposed cgMLST scheme for *P. aeruginosa* typed over 99% (2,314/2,325) of the isolates available for this study when we considered that at least 95% of all selected *loci* are present in the genome.

### cgMLST Analysis Compared With Core Genome SNP-Based Analysis for ST235

**FIGURE 4:**
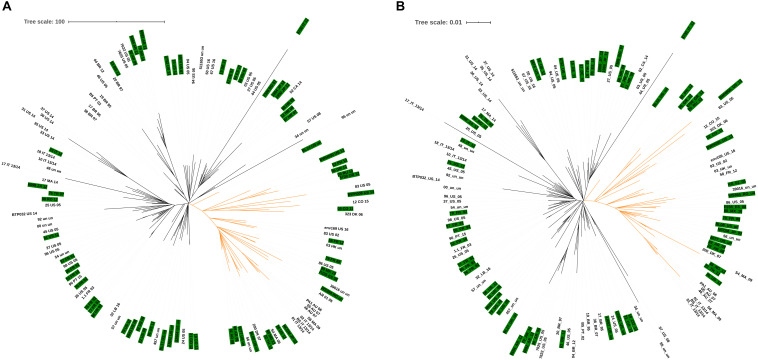
Similarity trees for *P.* aeruginosa ST235 isolates. **(A)** Similarity tree based on cgMLST analysis. **(B)** Similarity tree based on core genome SNP analysis. Branches in black and orange represent groups 1 and 2, respectively, previously described by Treepong et al. (2018). The 62 isolates previously evaluated by Treepong et al. are highlighted in green in the tree).

The cgMLST scheme was used to investigate the relatedness of *P. aeruginosa* isolates assigned to ST235. Using the proposed cgMLST scheme we were able to type all 128 ST235 isolates which remained after the genome quality assessment filters and an unrooted similarity tree was built based on the cgMLST gene targets proposed here (Figure 4A). Two major clusters are readily seen for these isolates, corroborating data previously shown by Treepong et al. (2018) when they evaluated 79 ST235 isolates using core genome analysis of SNPs. Of these 79 isolates, 62 were included in the cgMLST analysis and all clustered as originally proposed by Treepong et al. (highlighted with a green background in the tree). In fact, when we replicate the core genome typing approach proposed by Treepong et al. (using Parsnp for alignment) for the 128 isolates, the unrooted similarity tree reports that the isolates are clustered, identically, within two major groups (Figure 4B). This result indicates that the scheme proposed in this study may be used with the same purpose as core genome SNP analysis, with the advantage of using fewer genome regions.

### Comparison of Typing Resolution Between MLST and cgMLST

**FIGURE 5:**
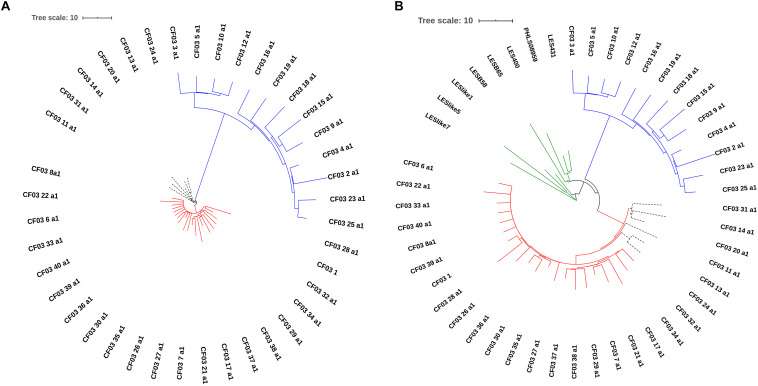
Similarity trees based on cgMLST analysis of ST146 strains. **(A)** ST146 *P. aeruginosa* Liverpool Epidemic Strain (LES) from a single cystic fibrosis patient (NCBI Bioproject PRJEB6642, *n* = 40). Two distinct clusters are highlighted in red and blue, and 6 more divergent isolates are indicated by dashed branches. **(B)** ST146 *P. aeruginosa* LES and 8 additional unrelated ST146 isolates from the United Kingdom and Canada. Blue and red branches represent the 2 clusters originally described for this set of isolates (dashed branches indicate the more divergent isolates) and branches in green represent the 8 additional isolates from United Kingdom and Canada.

A selection of isolates belonging to ST146 and ST274 was used to evaluate the typing resolution of the proposed cgMLST scheme. In order to determine epidemiological relationships between isolates, specific thresholds in cgMLST have been applied depending on the species. For instance, during outbreaks, isolates of *Listeria monocytogenes* (Ruppitsch et al., 2015), *Mycobacterium tuberculosis* (Kohl et al., 2014), *Legionella pneumophila* (Moran-Gilad et al., 2015) and *Klebsiella pneumoniae* have been shown to differ in fewer than 10 cgMLST alleles (Zhou et al., 2017). For *P. aeruginosa*, an *ad hoc* cgMLST scheme using SeqSphere+ (Ridom, Münster, Germany) considered fewer than 14 alleles for epidemiologically related isolates (Mellmann et al., 2016). The dataset for ST146 comprised 40 isolates of *P. aeruginosa* Liverpool Epidemic Strain (LES), collected from a cystic fibrosis patient during 2009 (Williams et al., 2015). Using a SNP-based analysis and a reference genome from an isolate collected in 1988 (LESB58), Williams et al. (2015) divided this set into two major lineages, A and B, with 13 and 27 isolates, respectively, separated by 79 SNPs. Our cgMLST-based analysis also proposed two major clusters, A and B, differing in 42 alleles (Figure 5A and Supplementary Figure S3). However, six isolates (dashed branches) were not grouped within the 2 major groups (Figure 5A). We do not have access to epidemiological data that could explain this separation. However, samples were collected during 2009 but were not associated with an outbreak, and, in this scenario, differences are not unexpected and could have arisen from microevolution. Eight additional ST146 isolates from the United Kingdom and Canada were added to this analysis, and they were clearly distinct from the LES isolates (Figure 5B). The minimum-spanning tree shows that the two clusters comprising the 40 LES and the 8 additional isolates differ in at least 25 alleles (Supplementary Figure S4).

**FIGURE 6:**
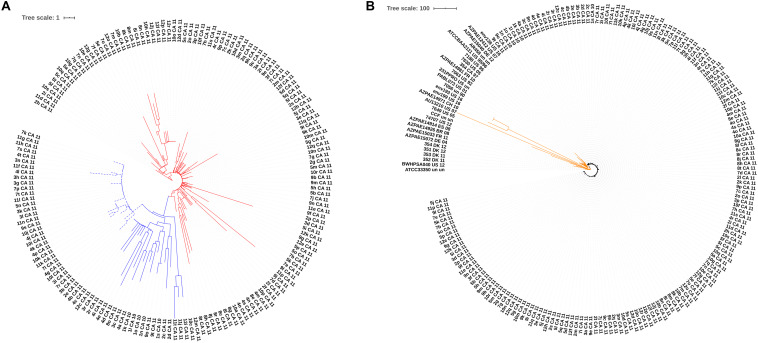
Similarity trees based on cgMLST analysis of ST274 isolates. **(A)** *P. aeruginosa* ST274 from a single cystic fibrosis patient (NCBI Bioproject PRJNA282164, *n* = 167). Blue and red branches represent clades A and B, respectively, corroborating data reported by Diaz Caballero et al. (2015). Dashed and solid branches identify subgroups in clade A. **(B)** *P. aeruginosa* ST274 isolates from different geographic regions were added to the dataset reported in **(A)**. Orange branches represent the 30 additional ST274 isolates from different geographic regions.

Another important strain associated with both colonization and infection in cystic fibrosis is ST274 (Fernández-Olmos et al., 2013; López-Causapé et al., 2013; Lopez-Causape et al., 2017). From a total of 197 available ST274 genome sequences, we were able to analyze all isolates using the proposed cgMLST scheme. Of these, 167 genome sequences belonged to *P. aeruginosa* isolates collected in Canada within 1 year and previously analyzed by Diaz Caballero et al. (2015) (NCBI Bioproject PRJNA282164, Supplementary Table S2), and 30 genome sequences were from unrelated isolates from different geographic regions (Brazil, Canada, China, Denmark, France, Germany, Spain, and United States). Diaz Caballero et al. (2015) separated the 167 isolates within two clades (A and B), comprising 55 and 112 isolates, respectively. The analysis based on the cgMLST gene targets for the 167 isolates also identified two major clades (Figure 6A and Supplementary Figure S5). However, within clade A, comprising 55 isolates reported by Diaz Caballero et al. (2015) cgMLST discriminates two subsets (dashed and solid branches). In order to understand the significance of differences within clade A, epidemiological data would be necessary. When adding 30 additional ST274 isolates to this dataset, cgMLST clearly identified the isolates belonging to the Diaz Caballero et al. study (Figure 6B), differing in fewer than 10 alleles, as expected, while the unrelated isolates differed in up to 662 alleles (Supplementary Figure S6). The cgMLST scheme was able to discriminate epidemiologically related isolates from study Diaz Caballero et al. (2015) from the unrelated isolates, all originally assigned identical STs.

Finally, we investigated the relatedness of 4 isolates involved in an outbreak of extended-spectrum β-lactamase SHV2a-producing *P. aeruginosa* ST235 in a previous study by Royer et al. (Royer et al., 2020) (NCBI Bioproject PRJEB32170). The authors used SeqSphere + software to create a cgMLST scheme comprising 4,162 gene targets. We used our scheme (2,653 *loci*) to evaluate the 4 isolates and report a very similar result: 0 to 2 alleles differed between the isolates (Supplementary Figure S7).

## Discussion

Simple, standardized, accurate and portable typing methods are needed for global surveillance programs and for outbreak investigations in clinical settings. Currently, PFGE and MLST are the two most frequently the used typing methods to investigate outbreaks (Maiden et al., 1998; Higgins et al., 2012). Most important drawbacks for these typing approaches include the low discriminatory power of MLST within outbreaks and difficulties for standardization of PFGE for inter laboratory comparisons (Higgins et al., 2012; Pérez-Losada et al., 2013).

Recent technical advances in WGS and lower costs have brought new possibilities for both global surveillance and local clinical investigations (de Been et al., 2015; Ruppitsch et al., 2015; Mellmann et al., 2016; Higgins et al., 2017; Nadon et al., 2017; Zhou et al., 2017; Janowicz et al., 2018; Kimura, 2018). Both wgMLST and cgMLST solutions are being implemented for a diverse set of microorganisms, such as those contemplated within the Global Platform for Pathogen Surveillance^6^. cgMLST is being considered the method of choice for typing pathogens for epidemiological surveillance of infectious diseases in the European Union and European Economic Area countries, overcoming SNP-based typing (Revez et al., 2017). However, there is no current consensus for a cgMLST scheme for *P. aeruginosa* despite two *ad hoc* cgMLST schemes previously proposed (Mellmann et al., 2016; Royer et al., 2020) (see section “Discussion” later).

cgMLST is a typing technique that relies on the availability of precisely sequenced full genomes for generating typing schemes. Using only CDs present in core genome, cgMLST reduces the number of sites typed in the analysis, when compared with core genome SNP-based analysis, and would also allow for a standardized, allele-based, reproducible nomenclature (Janowicz et al., 2018). For reliable results in cgMLST the quality of genome sequences is fundamental (Janowicz et al., 2018).

We propose a public cgMLST scheme for *P. aeruginosa* based on 130 whole genome sequences and a total of 2,653 target *loci* were validated in 2,184 unfinished genome sequences from diverse geographical regions, hosts, and human body sites. In our study we were able to successfully type over 99% of the evaluated genomes with a cgMLST scheme considering that at least 95% of the target *loci* were present. The initial number of candidate genes from full genome data was 3,168, and the reduction to 2,653 gene targets relates not only to the genomic variability of *P. aeruginosa*, but also to technical aspects: assembling issues such as gaps or incomplete assembling, inadequate coverage during sequencing with impact in consensus sequence quality, and absence of genes from the core genome. In all core-genome based methods, the size of the core genome inevitably decreases when the number of analyzed isolates increases (Denton et al., 2014; Van Tonder et al., 2014; de Been et al., 2015). The most commonly used metric for assessing assembling quality is N50 (Salzberg and Yorke, 2005; Yandell and Ence, 2012; Lantz et al., 2018). Although it is a popular metric, it is questionable and there is no consensus on an ideal N50 value for genomes: hard-hitting assemblers can produce large contigs when compared to conservative assemblers, but are more prone to errors (Salzberg and Yorke, 2005; Lischer and Shimizu, 2017; Lantz et al., 2018). Thus, in this study we chose to use lower number of contigs (< 200) for the selection of unfinished genomes for the validation of the proposed scheme.

During the scheme creation 11 fully assembled genomes were discarded due to lack of the candidate *loci*. Two of these sequences have been previously considered as outlier strains: PA7 (RefSeq assembly accession: GCF_000017205.1.) and CR1 (RefSeq assembly accession: GCF_003025345.2) (Roy et al., 2010; Sood et al., 2019). In the above-mentioned study by Sood et al., the authors evaluated 14 genomes considered as outliers and 64 classical *P. aeruginosa* genomes. When analyzed together, these sequences shared 2,885 core genome genes. However, when the 64 classical genomes were analyzed independently from the outliers, the number of common genes rose to 3,199, and the outliers alone shared 4,708 genes. When we look at the list of genomes we kept for the cgMLST scheme we see that the algorithm correctly discarded both PA7 and CR1 due to lack of target genes^7^ (see file analysis_cg/removedGenomes.txt and analysis_cg/GenomeRemoved120thr.txt). When using the determined set of cgMLST gene targets proposed here, 10 of the fully assembled genomes were successfully typed (contained at least 95% of the selected *loci*).

It has been proposed that an isolate should harbor at least 95% of the set of cgMLST gene targets (de Been et al., 2015; Ruppitsch et al., 2015; Ghanem and El-Gazzar, 2018). We were able to find 95% of our gene set in all but 11 genomes analyzed. Only one of the genomes lacking 95% of gene targets was considered a fully assembled sequence (RefSeq assembly accession: GCF_000473745.2). This isolate, *P. aeruginosa* VRFPA04, was collected in India from a human cornea sample (Murugan et al., 2016) and a more deep analysis of this sequence would be necessary to understand why these *loci* were not present. The unfinished sequences that had less than 95% of the targets did not belong to any particular origin or location.

Two *ad hoc* cgMLST schemes proposed for *P. aeruginosa* (Mellmann et al., 2016; Royer et al., 2020) used the commercial software Ridom SeqSphere+ (Ridom GmbH, Muenster, Germany) to generate the scheme. The above-mentioned schemes used the PAO1 genome as the seed genome and defined 3,842 and 4,162 target genes, respectively. In our scheme we propose 2,653 target genes, but we used 130 complete genomes as seed genomes. When we compare our gene targets to the one proposed by Mellmann et al. (2016) 2,081 of our gene targets (78.43%) match with the gene targets proposed by Mellmann et al. (data not shown). It is also important to mention that, in the literature, we find variation both in the total number of genomes included to define the core genome as well as parameters used for the delimitation of the core genome: sets between 4 and 1488 genomes have been used and between 665 and 5233 genes have been reported as constituting the core genome of *P. aeruginosa* (Valot et al., 2015; Vincent et al., 2017; Freschi et al., 2019; Sood et al., 2019; Subedi et al., 2019; Vasquez-Rifo et al., 2019). Besides the strict cut-off for the core genome gene selection used in this work (target *loci* were present in 100% of the 130 genomes at the creation of the scheme), other factors are worth consideration when comparing our gene set with that of Mellmann et al., we used a larger population for this study and a more varied one in terms of origin (human, ocean, animal, plants and industry) than the population they used, and we considered all unfinished genomes with acceptable quality at the validation phase, a factor that may have led to the exclusion of targets. For *K. pneumoniae*, similar results are seen, with two schemes, one proposing 1,143 gene targets and a second one 2,365 (Zhou et al., 2017; Miro et al., 2020), even though both used SeqSphere+ (Ridom, Münster, Germany) to generate the scheme. A third cgMLST scheme was proposed for *K. pneumoniae* using the open source software Bacterial Isolate Genome Sequence Database (BIGSdb) and 634 target genes were included in their cgMLST scheme (Bialek-Davenet et al., 2014).

We did not compare our scheme directly with what we would obtain with the same set of isolates and using SeqSphere+ (Ridom, Münster, Germany), but we evaluated isolates from an outbreak setting characterized using the software (Royer et al., 2020). The cgMLST scheme that we propose identified few allelic differences (0 to 2) between the isolates, a similar result to the one proposed by the authors, but using our reduced number of target genes when compared to theirs. Few differences were observed between isolates involved in an outbreak, an expected result due to limited time for intraoutbreak evolution (Ruppitsch et al., 2015; Zhou et al., 2017). For instance, in outbreak settings involving *L. monocytogenes* (Ruppitsch et al., 2015), *M. tuberculosis* (Kohl et al., 2014), *L. pneumophila* (Moran-Gilad et al., 2015) and *K. pneumoniae*, isolates have been shown to differ in fewer than 10 cgMLST alleles (Zhou et al., 2017). de Been et al. (2015) proposed an interpretation for cgMLST of *Enterococcus faecium*: (i) isolates differing from 0 to 20 alleles are considered undistinguishable or closely related, and possibly involved in an outbreak; (ii) isolates differing between 21 and 40 alleles possibly belong to the same outbreak; and (iii) and isolates differing in more than 40 alleles are unrelated. However, similarly to PFGE and MLST, thresholds should not substitute epididemiological investigation and, as previously suggested, microevolutionary events within each outbreak and the threshold of ≤10 different alleles warrant further validation (Ruppitsch et al., 2015; Zhou et al., 2017).

The cgMLST scheme proposed here helped to discriminate isolates belonging to the same ST, while clearly grouping isolates that had been epidemiologically linked in the original publications. Even though MLST is widely used in epidemiological studies, the fact that it addresses less that 0.1% of the genome, often limits its discriminatory power (Li et al., 2009; Sabat et al., 2013; Davis et al., 2015; Tang et al., 2017; Chen et al., 2018; Ghanem and El-Gazzar, 2018; Kimura, 2018). Our results are in agreement with recent publications for pathogens such as *Enterococcus faecium*, *L. monocytogenes*, *A. baumannii*, *K. pneumoniae*, and *E. faecalis* (de Been et al., 2015; Ruppitsch et al., 2015; Higgins et al., 2017; Zhou et al., 2017; Neumann et al., 2019).

Additionally, the cgMLST results presented here also corroborate with *P. aeruginosa* population studies for ST235 which used core genome SNP-based methodologies, with the clear advantage of targeting a smaller set of *loci* and the possibility of implementing a databank with unique profiles for *P. aeruginosa* typing based on its core genome (de Been et al., 2015; Ruppitsch et al., 2015; Ghanem and El-Gazzar, 2018; Pearce et al., 2018; Schürch et al., 2018; Neumann et al., 2019).

In conclusion we present a highly discriminatory cgMLST scheme for WGS-based typing of *P. aeruginosa* developed using an open access platform. Differently from the two available publications that used cgMLST to analyze *P. aeruginosa* isolates, we have made our scheme and associated files available at GitHub (https://github.com/BioinformaticsHIAEMolecularMicrobiolog y/cgMLST-Pseudomonas-aeruginosa) aiming to foment discussions and possibly help in establishing a cgMLST consensus for *P. aeruginosa*. Gene target lists and selected *loci* are available at this location. In the GitHub link we also present a step-by-step explanation on how new genome sequences may be analyzed. The remaining challenge is to establish an Internet-based nomenclature server to facilitate universal global nomenclature for any user, as currently available for MLST.

## Data Availability Statement

All datasets generated for this study are included in the article/Supplementary Material.

## Author Contributions

PS conceived and supervised the project. RS and RP performed the analysis with support from LM. RS, LM, and PS wrote the manuscript with contributions from BK. RS and PS interpreted the data.

## Conflict of Interest

The authors declare that the research was conducted in the absence of any commercial or financial relationships that could be construed as a potential conflict of interest.

## Footnotes

1 https://pubmlst.org/software/database/bigsdb/

2 https://github.com/BioinformaticsHIAEMolecularMicrobiology/cgMLST-Pseudomonas-aeruginosa

3 https://www.ncbi.nlm.nih.gov/assembly

4 https://github.com/sanger-pathogens/mlst_check

5 www.pubmlst.org

6 https://pathogen.watch/

7 https://github.com/BioinformaticsHIAEMolecularMicrobiology/cgMLST-Pseudomonas-aeruginosa
