# Supplementary material for: A Core Genome Multilocus Sequence Typing Scheme for Pseudomonas aeruginosa
Source: Front Microbiol. 2020 May 26;11:1049. doi: 10.3389/fmicb.2020.01049 (PMC7264379; doi:10.3389/fmicb.2020.01049)
Supplement: Supplementary file 1 [file Data_Sheet_1.pdf]

Supplementary Figures

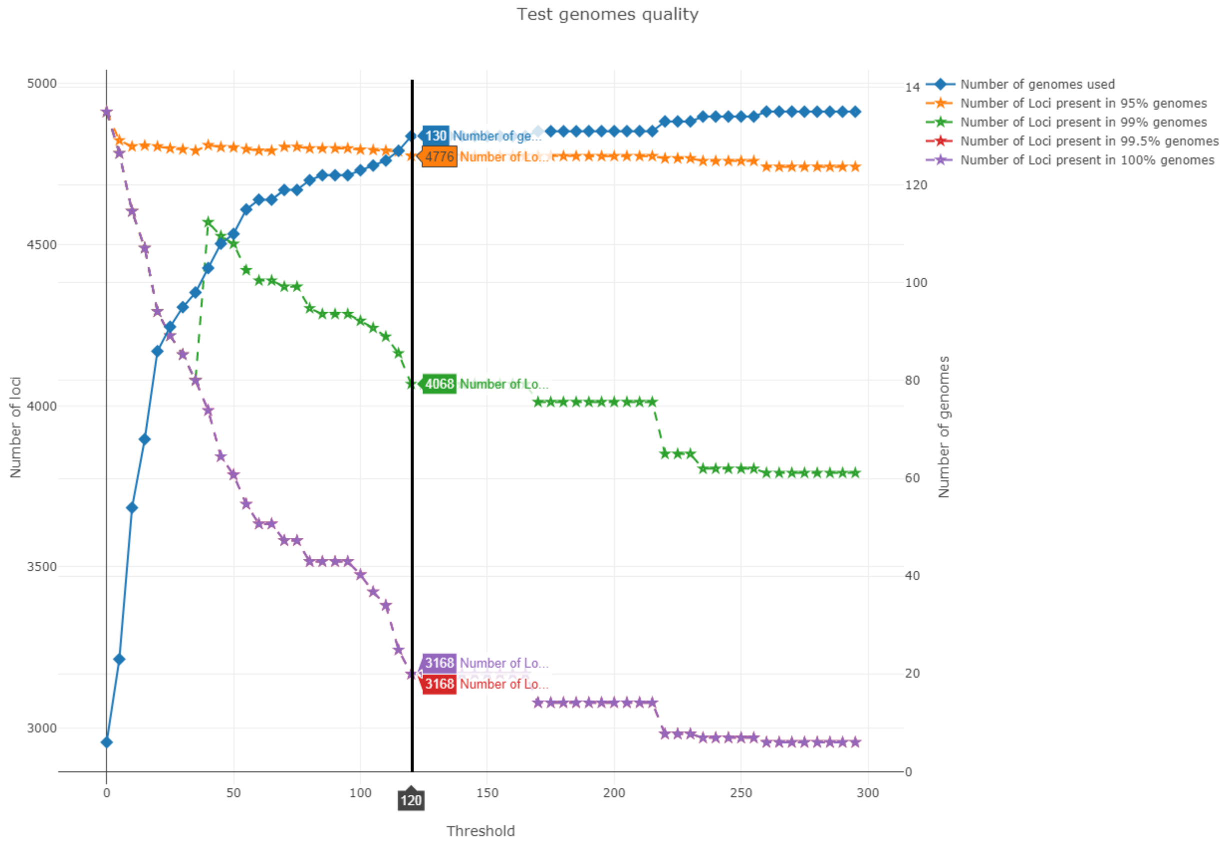

Figure S1. Number of *loci* and number of genomes in every exclusion threshold level for the 141 complete genomes that created the cgMLST scheme. The number of *loci* found in 95%, 99%. 99.5% and 100% of genomes are shown. The straight line represents the threshold that defined the maximum loss of *loci* by genomes.

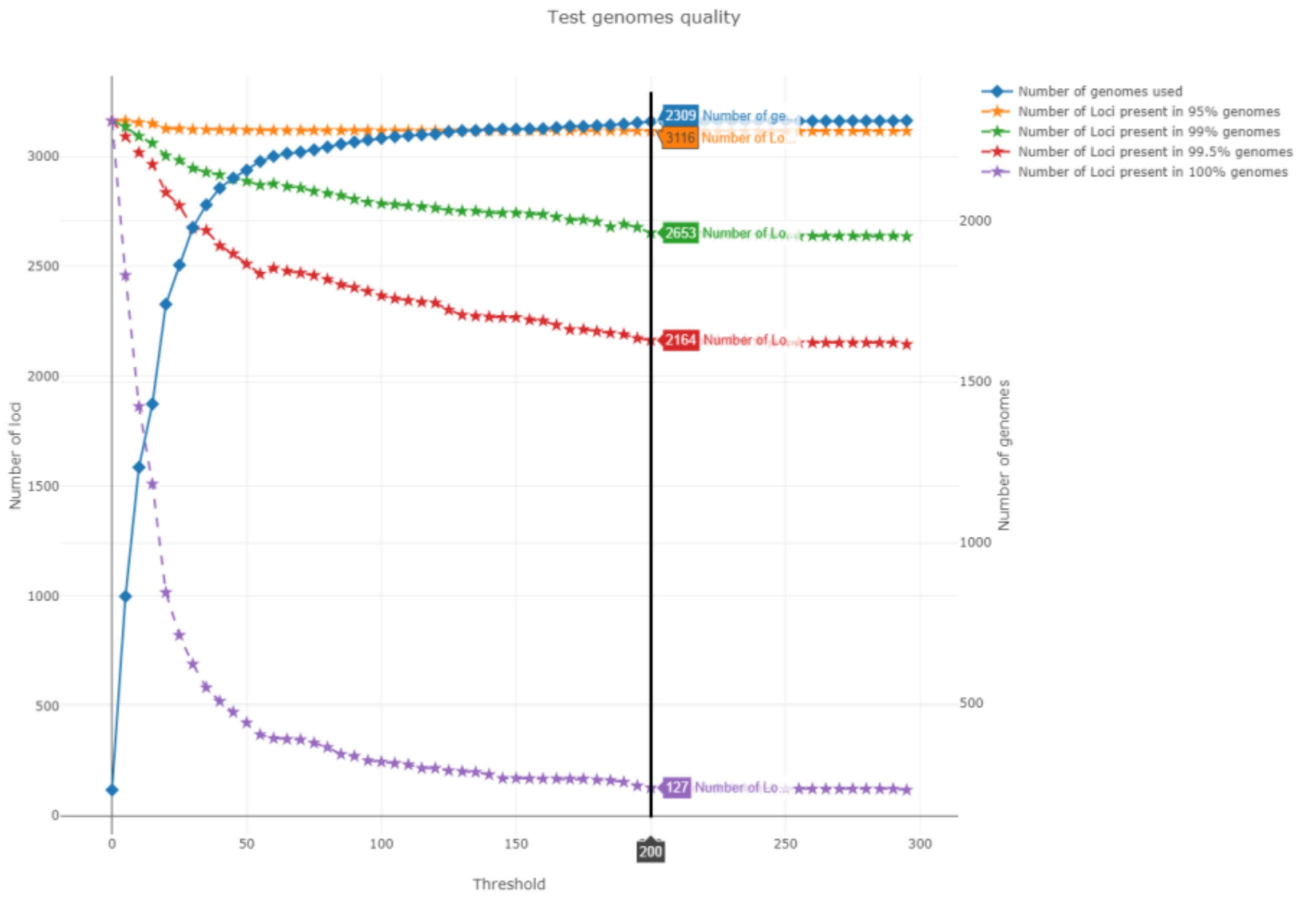

Figure S2. Number of *loci* and number of genomes in every exclusion threshold level for the 2184 validation genomes and 130 genomes that defined the cgMLST scheme. The number of *loci* found in 95%, 99% 99.5% and 100% of genomes are shown. The straight line represents the threshold that defined the maximum loss of *loci* by genomes.

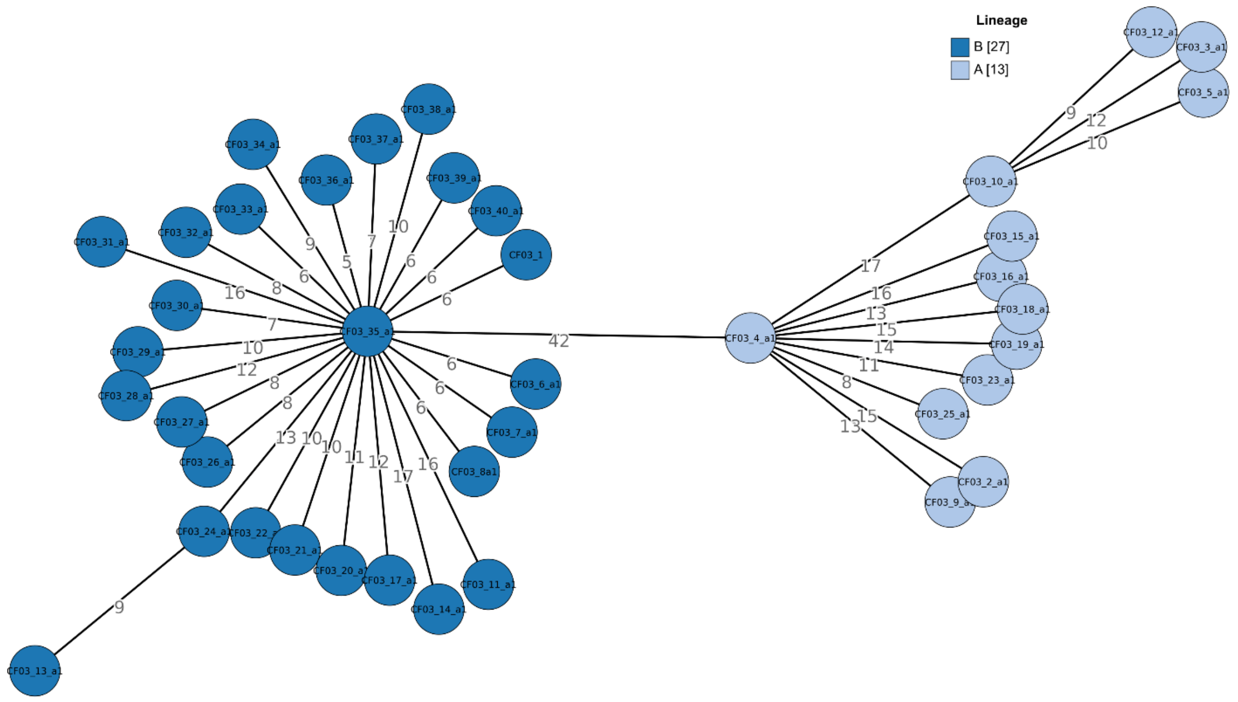

Figure S3. Minimum-spanning tree based on cgMLST allelic profiles of 40 *P. aeruginosa* isolates LES-146 evaluated by Williams et al., 2012 (NCBI Bioproject PRJEB6642). Each circle represents an allelic profile based on sequence analysis of 2,653 cgMLST target genes. The numbers on the connecting lines illustrate the number of target genes with differing alleles. The two lineages found both by Williams et al., 2015 and also by cgMLST distinguished by the colors of the circles.

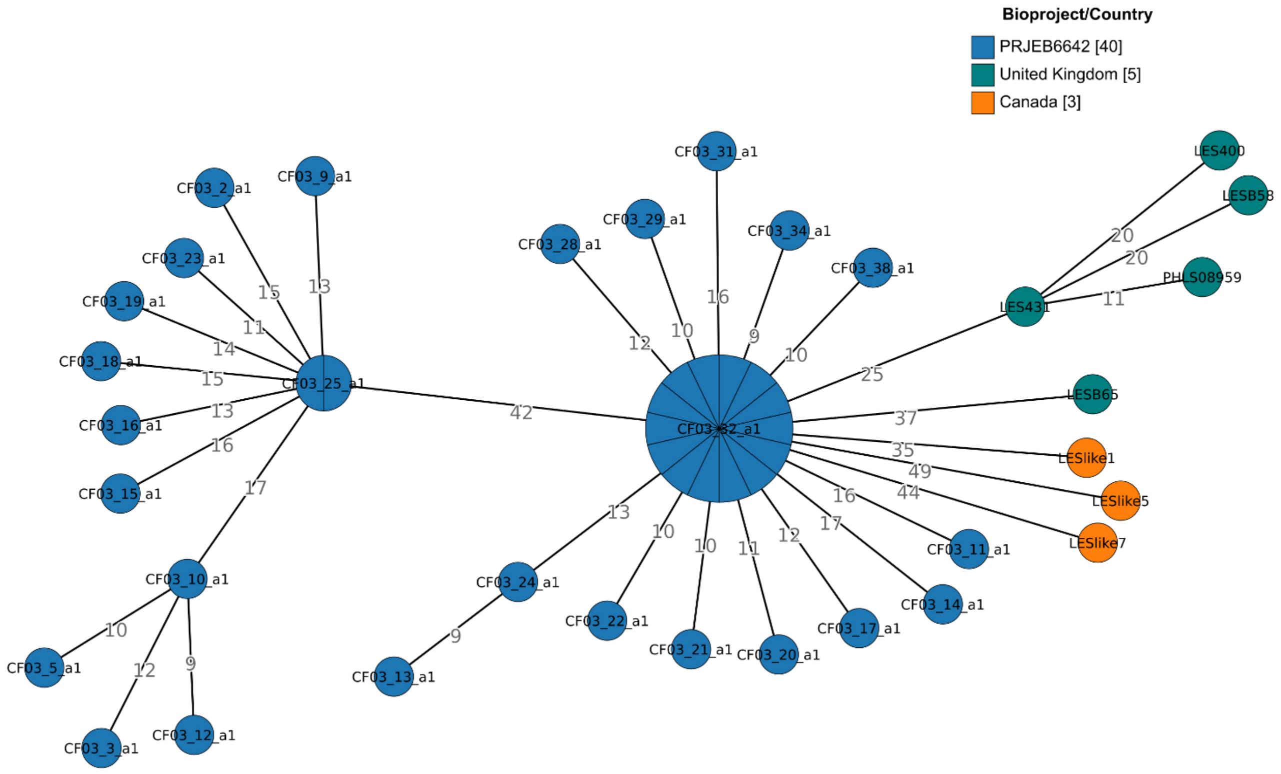

Figure S4. Minimum-spanning tree based on cgMLST allelic profiles of 48 *P. aeruginosa* ST146 isolates. Each circle represents an allelic profile based on sequence analysis of 2,653 cgMLST target genes. The numbers on the connecting lines illustrate the number of target genes with differing alleles. Isolates with a difference ≤8 target genes had their branches collapsed. The colors represent the different isolates according to the bioproject or country of origin. The 40 LES isolates in dark blue (NCBI Bioproject PRJEB6642) were evaluated by Williams et al., 2015.

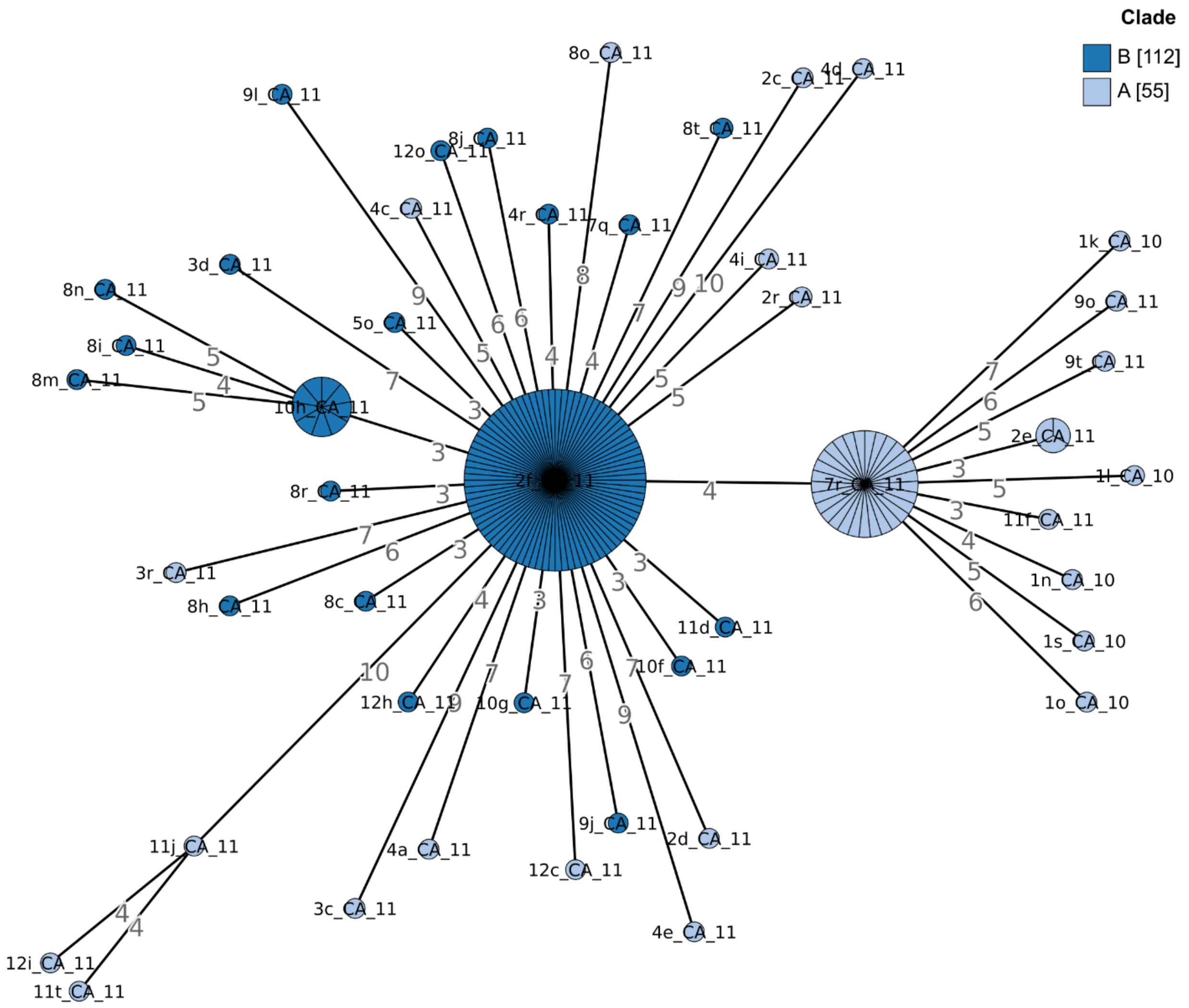

Figure S5. Minimum-spanning tree based on cgMLST allelic profiles of 167 *P. aeruginosa* isolates ST274 evaluated by Caballero et al., 2015. Each circle represents an allelic profile based on sequence analysis of 2,653 cgMLST target genes. The numbers on the connecting lines illustrate the number of target genes with differing alleles. Isolates with a difference ≤2 had their branches collapsed. The two clades found by Diaz Caballero et al., 2015 are distinguished by the colors of the circles.

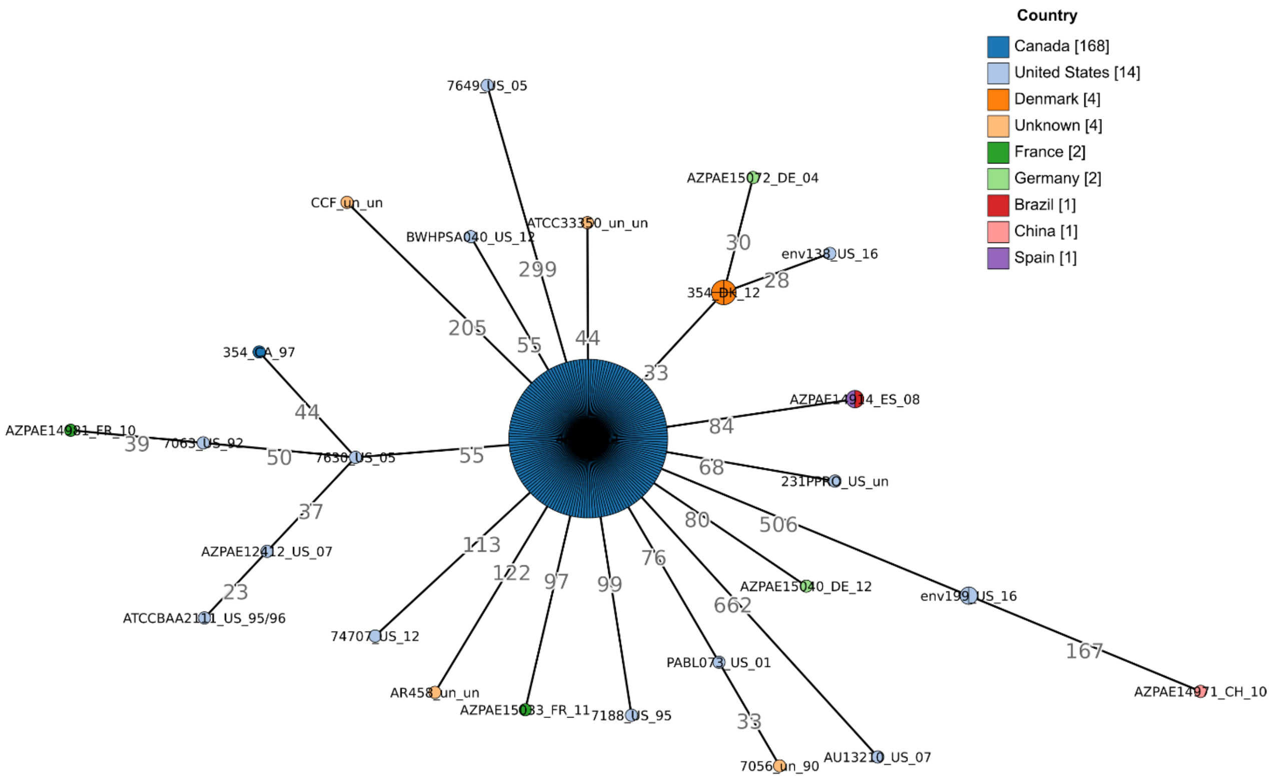

Figure S6. Minimum-spanning tree based on cgMLST allelic profiles of 197 *P. aeruginosa* ST274 isolates. Each circle represents an allelic profile based on sequence analysis of 2,653 cgMLST target genes. The numbers on the connecting lines illustrate the numbers of target genes with differing alleles. Isolates with a difference ≤10 alleles had their branches collapsed. The colors of the circles distinguish isolates according to the country of isolation. The 167 *P. aeruginosa* isolates ST274 evaluated by Diaz Caballero et al., 2015 are represented in the dark blue central circle.

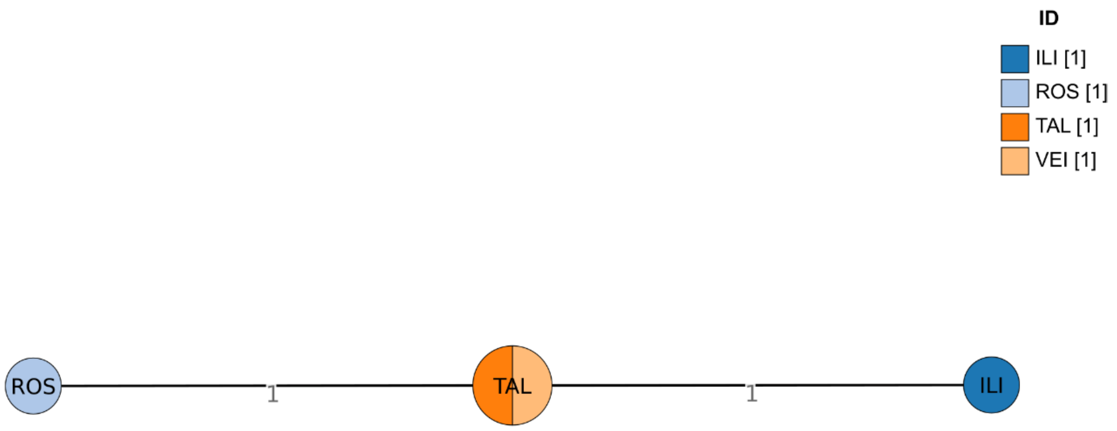

Figure S7. Minimum-spanning tree based on cgMLST allelic profiles of 4 isolates associated local outbreak of extended-spectrum β-lactamase SHV2a-producing *P. aeruginosa* ST235 evaluated by Royer et al., 2019 (NCBI Bioproject PRJEB32170). Each circle represents an allelic profile based on sequence analysis of 2,653 cgMLST target genes. The numbers on the connecting lines illustrate the numbers of target genes with differing alleles. The four isolates analyzed by cgMLST are distinguished by the colors of the circles.
